# Low-dose levels of bisphenol A inhibit telomerase via ER/GPR30-ERK signalling, impair DNA integrity and reduce cell proliferation in primary PBMC

**DOI:** 10.1038/s41598-017-15978-2

**Published:** 2017-11-30

**Authors:** Corinna Herz, Hoai Thi Thu Tran, Nina Schlotz, Karin Michels, Evelyn Lamy

**Affiliations:** 1 Institute for Prevention and Cancer Epidemiology, Molecular Preventive Medicine, Medical Center-University of Freiburg, Faculty of Medicine, University of Freiburg, Elsässerstraße 2, 79110 Freiburg, Germany; 2 Pharmaceutical Bioinformatics, Institute of Pharmaceutical Sciences, Albert-Ludwigs-University, Hermann-Herder-Str.9, 79104 Freiburg, Germany; 3 Institute for Prevention and Cancer Epidemiology, Medical Center-University of Freiburg, Faculty of Medicine, University of Freiburg, Elsässerstraße 2, 79110 Freiburg, Germany

## Abstract

Controversy exists about the human health risk of environmental exposure to bisphenol A (BPA). Telomerase activity is emerging both as biomarker and contributing factor for age-related diseases. The effects of BPA exposure at 1–1000 nM on telomerase, DNA integrity and cell proliferation were investigated in PBMC from human donors. Telomerase activity was determined by TRAP-ELISA assay and mRNA expression by qRT-PCR. Mechanistic studies were carried out on the ER/GPR30-ERK pathway using specific inhibitors/antagonists, the comet assay to quantify DNA damage and flow cytometry for cell proliferation. 24 h BPA exposure inhibited telomerase in a non-monotonic pattern with a peak inhibition of 32% at 1 nM (*p* ≤ 0.01). A significant telomerase inhibition was evident at 1 h after exposure with a minimum at 6 h. Elevated levels of DNA damage frequency and decrease in cell proliferation were evident upon long-term exposure. The results further demonstrate that BPA triggered rapidly an ER/GPR30-ERK transduction pathway that leads to decreased telomerase activity in human PBMC. This is the first study to demonstrate adverse impact of BPA at levels of current human exposure on telomerase in normal cells, mediated by ER/GPR30-ERK. The results suggest a potentially harmful influence of BPA on immune cells and should be addressed in future studies.

## Introduction

Bisphenol A (BPA) is a high production volume chemical (approximately 4.5 billion kilograms per year) used largely in the manufacture of polycarbonate plastics and as constituent of a wide array of consumer products, including plastic food containers and the lining of metal food cans^1^. BPA is also used as an additive in other types of plastics such as polyvinyl chloride (PVC) in medical tubing or water pipes and to make some dental sealants. Consequently, humans are ubiquitously exposed to the compound throughout the developed world mainly via dietary but also non-dietary sources^2^. So, unconjugated BPA was routinely detected in blood in the nanograms per milliliter (ppb) range (0.1–2 ng/ml or 0.4–8.8 × 10^−9^ M) in human blood biomonitoring studies^3^. Despite a wide body of research illustrating adverse effects of BPA, controversy still exists about the mechanisms of action of low-dose exposure, i. e. exposure within the range of chronic BPA levels of the typical human living in a developed country^4,5^. A recent literature review has found more than 90 studies linking BPA to human health^6^. It has been linked to pathologies in humans related to reproduction, development, cancer^7,8^ and immunological disorders^9^. Thus, recent studies show a link between pre- and post-natal exposure of BPA and increased risk for asthma and infections of respiratory tract in childhood^10,11^.

A hormonal mode of action of BPA is confirmed by *in vitro* experiments, which describe disruption of cell function at 10^−12^ M or 0.23 ppt. While BPA was initially considered to be a “weak” estrogen based on a lower affinity for estrogen receptor alpha (ERα) relative to estradiol (approximately 10.000-fold weaker)^12,13^, research now shows that BPA is equipotent with estradiol in its ability to activate responses via ERs associated with the cell membrane^14,15^. Additionally, BPA, in the nanomolar range, was shown to trigger signal transduction via the G protein-coupled receptor 30 (GPR30). This was then found to be transduced by the EGFR/ERK pathway and responsible for proliferation induction in both normal and cancer cells^16^.

Human telomerase has been identified as a new target of estrogen and its receptor^17^. In ER-positive MCF-7 breast cancer cells, estradiol activated telomerase activity. ERα bound to the estrogen response element (ERE) in the TERT promoter region in gel shift assays, and mutations in this element or tamoxifen exposure significantly reduced estrogen-induced TERT activation^17–19^. These findings are consistent with the hypothesis that telomerase activity is potentially under hormonal control in some estrogen-targeted tissues, such as the endometrium, the prostate and in epithelial cells with high renewal potential from estrogen-regulated tissues^20–22^ CD4(+) and CD8(+) T lymphocytes, B lymphocytes and NK cells contain intracellular ERα and ERβ receptors as estrogens are well-known regulators of the immune responses^23^. But, in human peripheral blood mononuclear cells (PBMC), results with estrogen are more inconsistent: at supra-physiological concentrations, estradiol increased telomerase mRNA expression and activity via ERα in one study^24^. However, in another study, no such regulation could be found^25^. Using high, non-physiologic BPA concentration of 1 µM, its ability to induce telomerase transcription in response to ER-binding was shown using an hTERT-luciferase promoter construct^26^. These findings suggest that BPA acts on hTERT in a manner similar to estrogen. So far, there are no reports investigating the impact of low-dose BPA on telomerase in normal human cells.

The results show a significant decrease in telomerase activity in activated primary human PBMC upon low-dose (1–10 nM) BPA exposure. This occurs by activation of ERK1/2 through ER/GPR30. Long-term cultured cells with multiple antigenic stimulations display increased DNA damage frequency and decreased cell proliferation upon continuous low-dose BPA treatment.

## Results

### Effect of low-dose BPA on telomerase of activated PBMC

**Figure 1:**
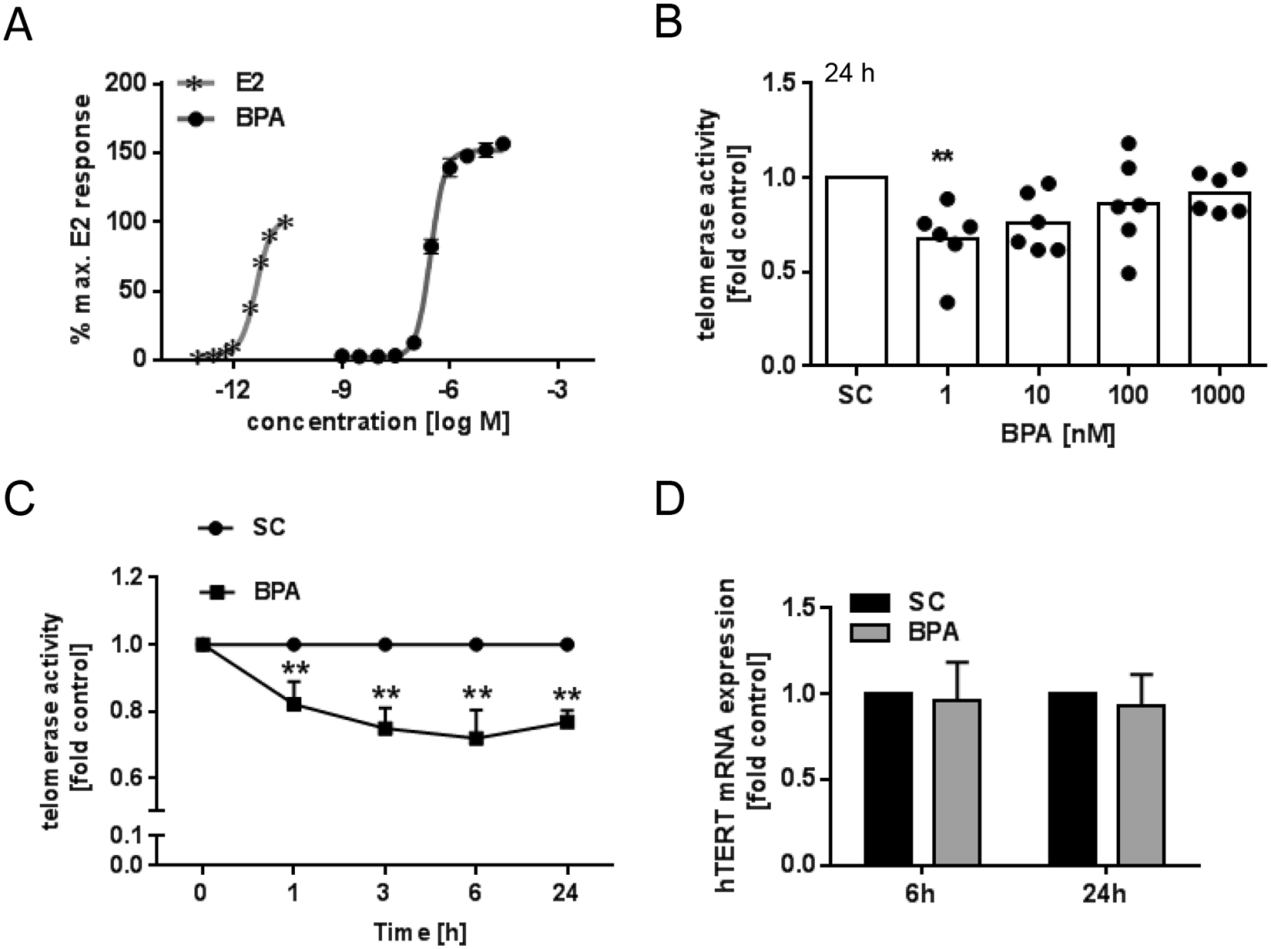
Effect of the estrogen active BPA on telomerase activity in activated PBMC. (**A**) Estrogenic activity of BPA was evaluated in comparison to E2 using the ER-CALUX^®^ reporter gene assay. The maximum response of E2 was set to 100%. Results are means, n = 3. (**B**,**C)** PBMC were stimulated with CD3/CD28 and treated with 1 nM BPA for the indicated time points. Telomerase activity was determined using the TRAP-ELISA assay. Results were calculated relative to the corresponding solvent control (SC, 0.1% DMSO). (**B)** Bars are mean values; each dot represents the result from one donor. (**C)** Results were presented as means + SD, n = 3. Significance of difference was calculated relative to the respective control, *p < 0.05; **p < 0.01. (D) Activated PBMC were analysed for full length hTERT mRNA expression after 6 h and 24 h exposure to 1 nM BPA using qRT-PCR. Porphobilinogen deaminase was used as reference gene. Results were calculated relative to the corresponding solvent control (SC, 0.1% DMSO) and are presented as means + SD. n = 6.

First, the estrogenic activity of BPA was evaluated in comparison to E2 using the ER-CALUX® reporter gene assay. As depicted in Fig. 1A, the detected estrogenic potency was E2 ≫ BPA with calculated EC_50_–values of 4 × 10^−12^ M and 2.87 × 10^−7^ M, respectively. We used a physiologically relevant approach with functional active antibodies to CD3 and CD28 to activate T cells in a manner that partially mimics activation by antigen-presenting cells. Therefore, BPA was investigated in CD3/CD28-activated PBMC. As depicted in Fig. 1B, BPA repressed telomerase activity in PBMC in a non-monotonic pattern. At a concentration of as low as 1 nM telomerase was suppressed by 32%; increasing concentrations gradually abolished the inhibitory effect. To gain insight into the time kinetics of telomerase suppression by BPA, analysis was carried out for 1 to 24 h (see Fig. 1C). Already within 1 h, the inhibitory effect of 1 nM BPA on telomerase enzyme activity became evident. This effect was not due to a change in hTERT mRNA expression, as shown in Fig. 1D, for 6 h or 24 h treatment with 1 nM BPA.

### Effect of long-term BPA exposure on telomerase, DNA damage and cytokine release

**Figure 2:**
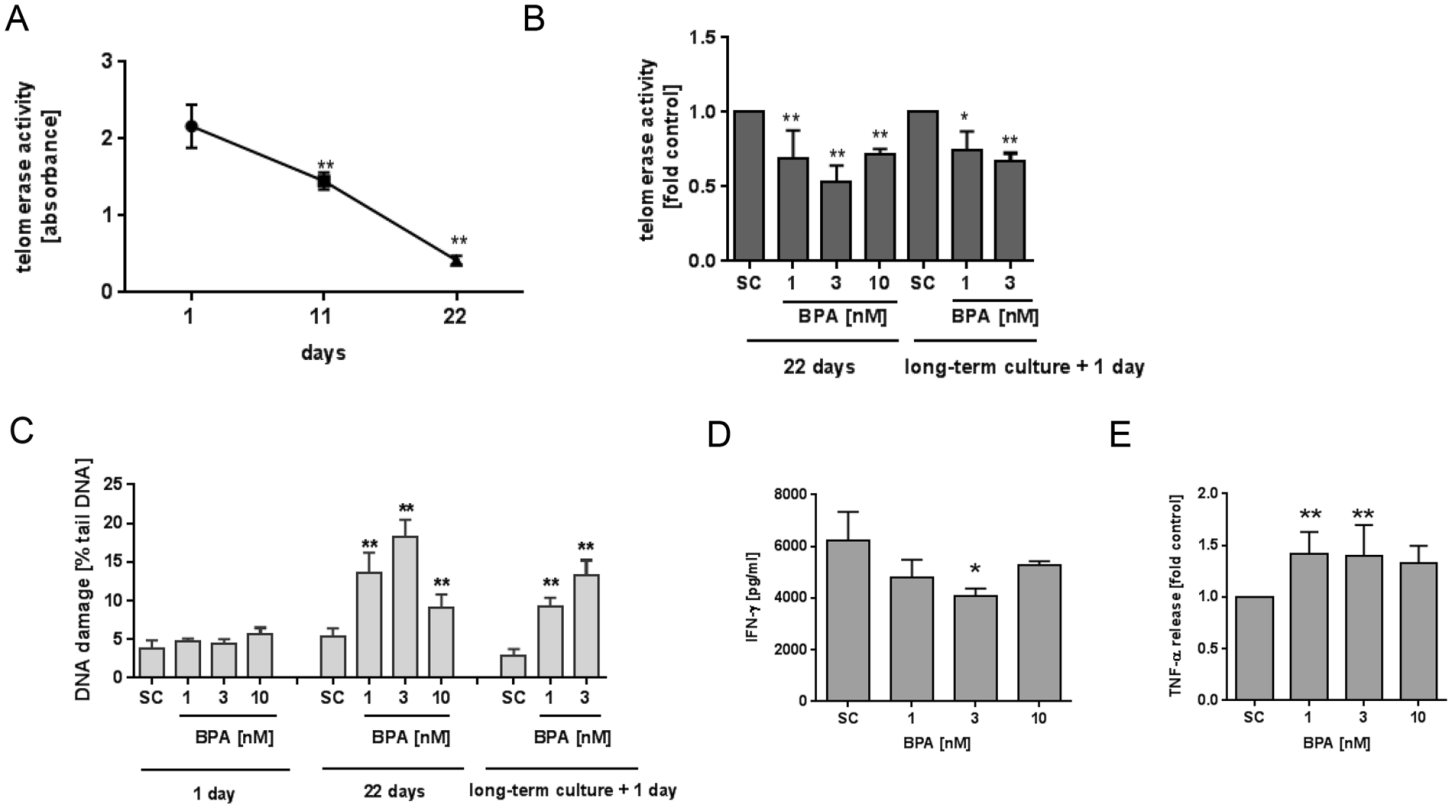
Effect of long-term BPA exposure on telomerase activity and DNA damage induction. Isolated PBMC were cultured for the indicated time points using CD2/CD3/CD28 beads and IL-2. Telomerase activity was determined using the TRAP-ELISA assay. (**A)** Change in telomerase activity of untreated cultured cells over time (n = 3). (**B)** Cells were cultured for three weeks either under constant BPA/solvent exposure or exposed to BPA/solvent at day 21 for 24 h and prepared for TRAP-ELISA assay (n ≥ 3). (**C)** Cells were cultured for three weeks either under constant BPA/solvent exposure or exposed to BPA/solvent at day 21 for 24 h and prepared for DNA damage analysis by the comet assay (n ≥ 3). Cells were cultured for 25d under constant BPA/solvent exposure and prepared for analysis of IFN-γ (**D**) (n ≥ 2) or TNF-α release (**E**) (n ≥ 3) using ELISA kits. Results are mean values + SD. *p < 0.05; **p < 0.01. Significance of difference was determined compared to the respective solvent control. SC: solvent control, 0.1% DMSO.

To analyze the effect of long-term BPA exposure on cells, PBMC were treated with solvent or BPA and stimulated with CD2/CD3/CD28 beads and IL-2 for three weeks. Cell characteristics are given in representative scattergrams in Supplement Figures S1 and S2. As can be seen in Fig. 2A, the capacity of cells to activate telomerase upon re-stimulation decreased with time (70% at day 11 and 20% at day 22). The observed repressive effect of BPA on telomerase activity was not transient; after 22 days of continuous exposure, a mean inhibition of 36% and 50% by 1 nM and 3 nM BPA was detected, respectively. This effect was partly abolished at higher concentrations. Long-term cultures with already diminished telomerase activity were still sensitive to the effect of BPA exposure. Enzyme activity was reduced by 25% (1 nM) and 33% (3 nM) upon 24 h treatment as compared to control cells (see Fig. 2B).

Next, the effect of BPA on DNA damage induction was studied. DNA damage was quantified after short term (24 h) BPA exposure and compared to the results derived by long-term (22 days) exposure. As depicted in Fig. 2C, no change in DNA damage frequency, as determined by the comet assay, could be detected after 24 h exposure to low concentrations of BPA. However, after 22 days of continuous exposure, a significant increase in DNA damage became evident at 1–3 nM. As with telomerase, further increase in BPA diminished the effect on DNA damage again. Long-term cultures, exposed to BPA for 24 h, also demonstrated elevated sensitivity to DNA damage induction by BPA. This was significant already at 1 nM BPA.

The relevance of our observations was further studied using IFN-γ and TNF-α release from long-term cultured cells. As given in Fig. 2D, IFN-γ release was reduced upon BPA treatment; in Fig. 2E it is shown that TNF-α release was increased upon BPA treatment. Then again, the dose-response curves were non-monotonic in the range between 1 to 10 nM, as already observed for the other parameters.

### Effect of long-term BPA exposure on cell proliferation

**Figure 3:**
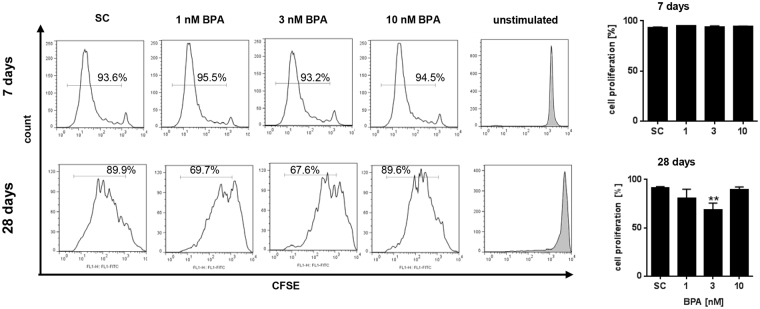
Effect of long-term BPA exposure on cell proliferation. Isolated PBMC were cultured using CD2/CD3/CD28 beads and IL-2. Cell proliferation was quantified using CFSE staining after 7d and 28d. Representative histograms are shown (left) and summarized in graphs (right). Results are means + SD (n = 3). SC: solvent control 0.1% DMSO; *p < 0.05; **p ≤ 0.01. Significance of difference was determined compared to the respective solvent control.

We next studied the effect of BPA on cell proliferation using CFSE staining. After 7d of exposure, no effect could be seen at all concentrations tested (see Fig. 3, upper panels). However, continuous BPA exposure for 28d resulted in a deceased proliferation rate at a concentration between 1 to 3 nM. At 10 nM, this effect was abrogated again (see Fig. 3, lower panels).

### Relevance of the ER/GPR30-ERK pathway for telomerase inhibition by BPA

**Figure 4:**
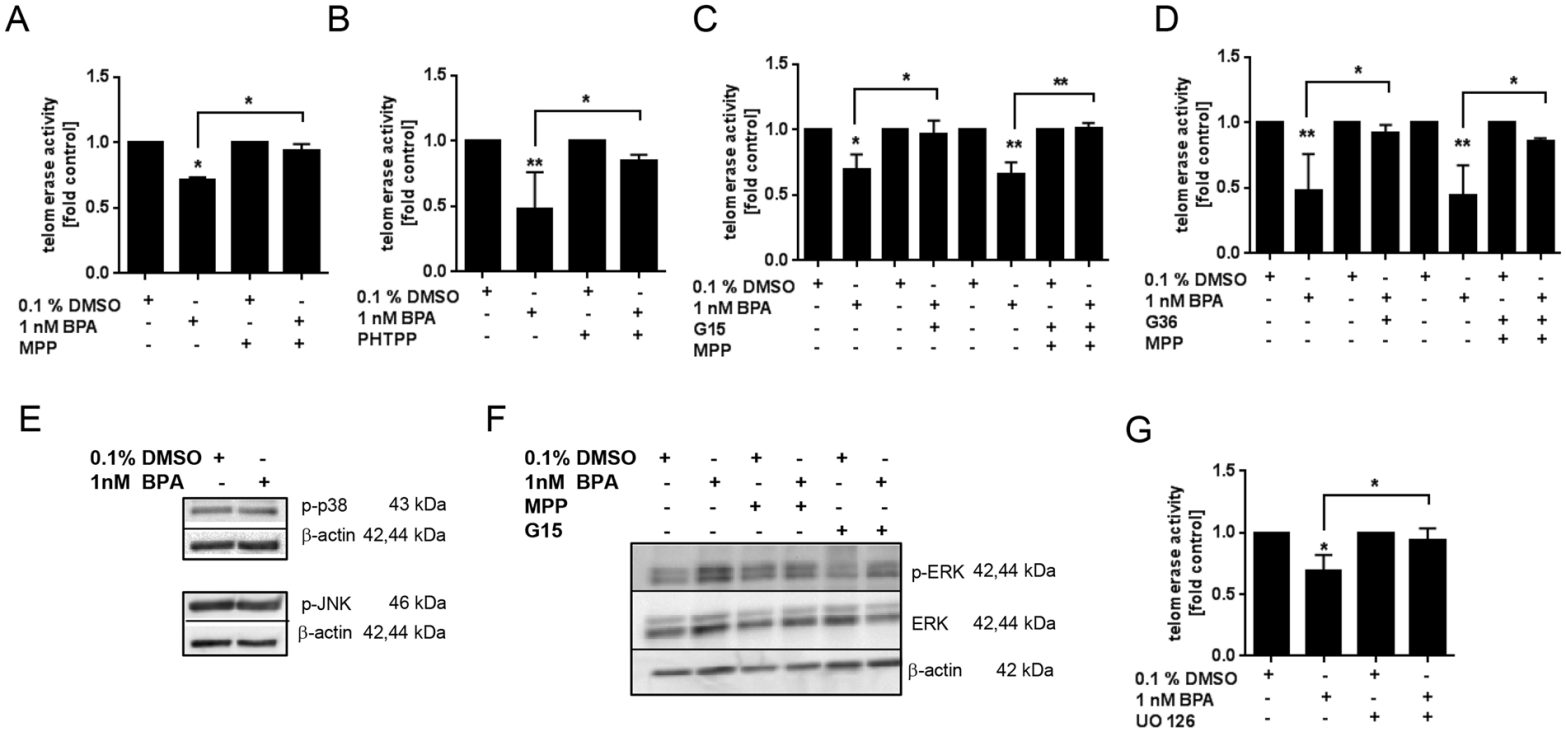
Effect of BPA exposure on ER/GPR30-ERKpathway. (**A–D**,**F)** PBMC were stimulated with CD3/CD28 and treated with solvent control or 1 nM BPA alone or in combination with antagonists/inhibitors for 6 h; ERα antagonist (100 nM MPP) **(A)**, ERβ antagonist (150 nM PHTPP) **(B)**, GPR30 antagonist (10 nM G15) **(C)** or 100 nM G36) **(D)**, ERK1/2 inhibitor (10 nM UO126) **(G)**. Telomerase activity was determined using the TRAP-ELISA assay and calculated relative to the respective solvent control (SC, 0.1% DMSO). Results are means + SD of at least 3 independent experiments. *p < 0.05, **p < 0.01; significance of difference was determined compared to the respective solvent control. (**E and F)** PBMC were treated to 1 nM BPA for 15 min and total lysate was subjected to immunoblotting. The pictures depict representative immunoblots of phosphorylated p38 and JNK (**E**) or phosphorylated ERK1/2 and whole protein ERK1/2 (**F**). β-actin was used as loading control.

To investigate whether the BPA-induced telomerase inhibition is mediated by the ER, we used the ERα-specific inhibitor MPP as well as the ERβ-specific inhibitor PHTPP. Upon treatment of PBMC with MPP (Fig. 4A), or PHTPP (Fig. 4B) telomerase inhibition by BPA was significantly abolished. In a next step, PBMC were treated with the GPR-selective antagonist G15 to elucidate the involvement of GPR30 in signal transduction. Treatment with either G15 alone or in combination with MPP completely abrogated the inhibitory effect of BPA on telomerase activity (see Fig. 4C). This result was confirmed using another GPR-selective antagonist (G36) (see Fig. 4D). Then, we investigated the relevance of MAPK activation for downstream signalling by BPA. As illustrated in Fig. 4E, the activation of neither p38 nor JNK was effected by BPA. In contrast, BPA activated ERK1/2 in PBMC (Fig. 4F). The MAPK ERK1/2 is a known downstream event of ERα/GPR30 induced by BPA^27^ and here we could also show that ERK activation was blocked by either MPP or G15 (Fig. 4F). The relevance of ERK1/2 activation for telomerase inhibition by BPA was then confirmed using the specific ERK inhibitor UO126, which indeed prevented the action of BPA on telomerase (see Fig. 4G).

## Discussion

By now, numerous clinical studies link shorter telomere length and lower telomerase activity with age-related diseases and earlier mortality^28,29^. Telomerase activity is thought to be dynamic and is likely an essential modifiable factor in mediating environmental and lifestyle factors^28^. Ectopic hTERT expression in human T cells results in lifespan extension in culture, which underlines the importance of telomerase in T cell function^30,31^. In light of increasing elderly populations in Western societies, it is critical that we understand whether exposure of BPA could negatively affect normal cells from the human immune system and consequently add to diseases such as cancer, infections or autoimmune disorders in man. The present *in vitro* study provides now important evidence that BPA is hormonally active on telomerase which was used as key readout for the effects of low-dose BPA exposure on human PBMC.

In order to carry out their physiological functions, immune cells require many cell divisions. When T cells are stimulated through their T cell antigen receptor, they are able to upregulate telomerase activity^32^ which is critical, since low telomerase activity has been shown to lead to a premature decline of the immune system. This is, however, a transient event and telomerase decreases significantly with increasing rounds of cell division^33,34^. We found that BPA at concentrations as low as one nM significantly reduced the activity of telomerase during primary stimulation in human PBMC. Importantly, the inhibitory potential was persistent during subsequent stimulation of previous activated cells. This observation was paralleled by reduced cell proliferation and increased DNA damage frequency as compared to control. Further, BPA treatment also resulted in an increase in TNF-α release in long-term cultured cells. These observations bring up the concern, that BPA may be harmful for physiologic situations requiring enhanced immune cell telomerase activity or that BPA eventually in consequence promotes conditions of immune impairment. Features of this include accumulation of cells with reduced proliferation capacity and telomerase activity as well as enhanced secretion of inflammatory cytokines^35,36^. Recently, an inverse association between age and DNA repair activity of damaged DNA was demonstrated in human lymphocytes^37^ and reduced DNA mismatch repair capacity, which is capable of rectifying errors in DNA replication found in T cells^38^. Here for example, telomerase is essential for the repair of telomeric DNA following damage by oxygen radicals^39^. A decrease in the anti-viral cytokine IFN-γ, as observed upon BPA long-term treatment in the present study, is also known to impair the ability of immune cell function to act against virus infected cells^35,40^.

Telomerase regulation happens by control of hTERT transcription, alternate splicing of hTERT, and assembly of telomerase holoenzyme^41^. BPA has been reported to induce telomerase transcription in human MCF7 breast cancer cells in response to ER-binding, but only at a non-physiologic concentration of one µM^26^. The same transcription potential was reached with E2 at a concentration of 1000-fold less^12,13^. In the present study we found no effect of BPA on hTERT mRNA expression at the concentration of one nanomolar and thus conclude that BPA impacts telomerase direct at enzyme activity level. Further, exposure to BPA caused rapid activation of the MAPK ERK1/2 via both ER and also GPR30. Blocking either ER or the GPR30 receptor suppressed both phosphorylation of ERK and BPA-mediated telomerase inhibition. Using the ERK1/2 specific inhibitor UO126 confirmed that this effect is signalled through the MAPK pathway. It has been proposed earlier that GPR30 might act as “collaborator” of membrane ER, as crosstalk between both receptors has been described^42^ and this could be true for telomerase inhibition by BPA.

In conclusion the present study provides new insight towards effects of BPA at levels of current human exposure in that significant telomerase inhibition in activated human PBMC is demonstrated and this is mediated by an ER/GPR30-ERK signalling. Elevated DNA damage frequency and decreased proliferation rate suggest a negative influence of BPA on immune cells upon low-dose long-term treatment and should be addressed carefully in future studies. Further, for most of the observations reported here, a non-monotonic dose response curve (NMDRC) was evident indicating that these effects are not predicted by effects at higher doses. Until today NMDRCs have been demonstrated for natural hormones and endocrine disrupting chemicals in a variety of biological systems including cultured cells and human populations. For BPA *in vitro* literature illustrated that NMDRCs occur in more than 1/5 of experiments^4^. In general, there are several mechanistic hypotheses to explain for these NMDRCs of EDR including specific receptor affinity or desensitization, catabolization of substances, formation of mixed-ligand dimers or other unknown factors^43^. The reason for the NMDR of BPA on telomerase will be subject for further research. Given detectable BPA levels in 95% of the population and continued exposure due to the ubiquitous presence of BPA in our environment the findings may have considerable implications for chronic disease susceptibility and longevity.

## Material and Methods

### Materials

DMSO (purity > 99%), Acrylamide (30%, Mix 37,5:1) and bovine serum albumin (BSA) fraction V were from Applichem GmbH (Darmstadt, Germany). β-mercaptoethanol were purchased from Fluka (Buchs, Switzerland). Dulbeccos Minimal Essential Medium (DMEM), fetal calf serum (FCS), trypsin 10x (25 mg/ml), trypsin-EDTA 10x (5 mg/ml, respectively 2.2 mg/ml), L-glutamine and phosphate buffered saline (PBS, without Ca and Mg), Penicillin-Streptomycin (P/S) solution and RPMI-1640 were from life technologies Invitrogen (Darmstadt, Germany). Fetal calf serum (FCS) charcoaled stripped was from Biochrom (Berlin, Germany), nuclease free water from Qiagen (Hilden, Germany). Bisphenol A (>99%), Tween-20, protein standard BSA and Ammoniumpersulfat were purchased from Sigma-Aldrich Chemie GmbH (Taufkirchen, Germany). Anti-human CD3 and CD28 functional grade purified antibodies were from eBioscience affymetrix (Frankfurt, Germany). Recombinant human Interleukine-2 (IL-2) was purchased from Miltenyi Biotec GmbH (Bergisch Gladbach, Germany). Other reagents were obtained as follows: MPP dihydrochloride (1,3-Bis(4-hydroxyphenyl)-4-methyl-5-[4-(2-piperidinylethoxy)phenol]-1H-pyrazole dihydrochloride) and PHTPP (4-[2-Phenyl-5,7-bis(trifluoromethyl)pyrazolo[1,5-a]pyrimidin-3-yl]phenol) from Santa Cruz Biotechnonoly (Heidelberg, Germany), UO126 from New England BioLabs, GmbH (Frankfurt, Germany), G15 (4-(6-Bromo-1,3-benzodioxol-5-yl)-3a,4,5,9b-3*H*-cyclopenta[*c*]quinoline) and G36 (( ± )-(3aR*,4 S*,9bS*)-4-(6-Bromo-1,3-benzodioxol-5-yl)-3a,4,5,9b-tetrahydro-8-(1-methylethyl)-3H-cyclopenta[c]quinoline) from Tocris Bioscience (Wiesbaden-Nordenstadt, Germany). Amersham™ ECL Select™ and Hybond ECL Nitrocellulose Membrane were obtained from Ge Healthcare Biosciences AB (Uppsala, Sweden), Quick Start Bradford 1x Dye Reagent from BioRad Laboratories GmbH (Munich, Germany) and Page Ruler Plus Prestained Protein ladder from Thermo Fisher Scientific (Waltham, Massachusetts, USA). The following primary antibodies were used for immunoblotting: anti-p-ERK1/2 (Thr202/Tyr201, 1:2000), anti-ERK1/2 (1:2000, clone L34F12), anti-p-p38 (Thr180/Tyr182, 1:1000) and anti-p-JNK (Thr183/Tyr185,1:1000) from Cell Signalling (Danvers, Massachusetts, USA), and anti-beta-actin (1:10000, clone AC-74) from Sigma-Aldrich Chemie GmbH (Taufkirchen, Germany). The horseradish peroxidase (HRP)-labelled secondary antibodies anti-mouse and anti-rabbit were purchased from Cell Signalling (Danvers, Massachusetts, USA). Lysis buffer and illuminate mix for the ER-CALUX® assay were purchased from BioDetection Systems (Amsterdam, The Netherlands).

### Isolation of human PBMC from volunteers’ blood

PBMC were isolated from fresh blood obtained from volunteers at the University of Freiburg - Medical Center after written informed consent. The study was approved by the ethics committee of the University of Freiburg and carried out according to their guidelines and regulations. At the time of blood sampling, volunteers (male and female donors, aged between 22 to 42 years) had a normal BMI, were healthy and did not take any medication including contraceptives. All experiments and methods were conducted according to good laboratory practice. PBMC were isolated within 2 h by centrifugation on a LymphoPrepTM gradient (density: 1.077 g/cm^3^, 20 min, 500xg,) were then washed twice with PBS and cell viability and concentration was determined using the trypan blue exclusion test. PBMC (1 × 10^6^ cells/mL) were cultured in phenol red free RPMI 1640 medium supplemented with 5% heat-inactivated fetal calf serum (charcoal-treated), 2 mM L-glutamine, 100 U/mL penicillin/streptomycin at 37 °C in a humidified incubator with a 5% CO_2_/ 95% air atmosphere. Cells were exposed to 0. 1% DMSO (solvent control) or 1–1000 nM BPA.

### Long-term culture of PBMC

Long-term culture of PBMC was carried out as described before^44^. Cell suspensions were counted using trypan blue and splitted every three to four days. Cells were maintained at a concentration of 1 × 10^6^ cells/mL in fresh culture media supplemented with 20 U/mL IL-2 and the respective test compounds (BPA at 1, 3 and 10 nM). Each 10 days, cells were re-stimulated with CD2/CD3/CD28 beads (T Cell Activation/Expansion Kit, human, Miltenyi Biotec, Bergisch Gladbach, Germany). Aliquots were taken on a regular basis from each cell culture for determination of parameters.

### Analysis of cell proliferation using CFSE staining

For proliferation analysis, cells cultured for different time points were washed once with PBS and then resuspended in PBS supplemented with 5% heat inactivated, charcoaled treated FCS. This was followed by incubation for 10 min at RT with 5 µM 5(6)carboxyfluorescein diacetate succinimidyl ester (CFSE; eBioscience, Frankfurt, Germany). Culture medium was added and cells incubated for 5 min on ice before washing with medium two times. Cell proliferation was analysed on day 7 after staining using flow cytometry using a FACSCalibur™ (BD Biosciences, Heidelberg, Germany).

### ER-CALUX^®^ reporter gene assay

Estrogen receptor (ER)-mediated estrogenicity was determined using the ER-CALUX® reporter gene assay. The T47Dluc cells used for this assay contain both ERα and ERβ and were obtained through a license agreement with BioDetection Systems (Amsterdam, The Netherlands). The assay was performed according to the standard operating procedure of BDS, which is based on and modified from Legler *et al*.^45^. In brief, cells were seeded in 96-well microtiter plates at a density of 1 × 10^4^ cells per well. After 48 h, cells were exposed to BPA (1 nM–100 µM) for 24 h. An estrogen standard dilution series (0.1 pM–30 pM) was included. Cells were lysed and luciferase activity was measured after adding 100 µl illuminate mix using a luminometer (Infinite M200, Tecan, Männedorf, Switzerland). Substances were tested in triplicate in at least three independent experiments.

### Telomerase activity measurement by TRAP-ELISA assay

Telomerase activity was detected using the *TeloTAGGG* Telomerase PCR ELISA Kit, from Roche (Mannheim, Germany) as described before^41^.

### Protein analysis by immunoblotting

Proteins were analysed by immunoblotting as described before^41^.

### qPCR of full length *hTERT* transcript

Reverse transcription and qPCR of full length *hTERT* transcript was performed as described before^41^.

### Single cell gel electrophoresis (SCGE) assay

The SCGE assay, also known as comet assay, was carried out as described earlier^46^. The % Tail DNA was calculated as indicator of DNA damage.

### Quantification of cytokine release by ELISA Assay

To analyze for release of IFN-γ or TNF-α, **s**upernatants were used for photometric quantification by the human IFN-γ ELISA Ready-Set-Go kit and the TNF-α ELISA kit, respectively, both supplied from eBioscience (Frankfurt, Germany) according to the manufacturer’s instructions.

### Statistics

Results were analyzed using GraphPad Prism 6.0 software (La Jolla, California, USA). Data are presented as means + SD. Statistical significance was determined by the ordinary one-way ANOVA followed by Bonferroni correction or Kruskal-Wallis test. *P* values < 0.05 (*) were considered statistically significant and < 0.01 (**) were considered highly statistically significant.

### Data Availability

All data generated or analyzed during this study are included in this published article (and its Supplementary Information files).

## Electronic supplementary material

Supplementary information
