# Supplementary material for: Low-dose levels of bisphenol A inhibit telomerase via ER/GPR30-ERK signalling, impair DNA integrity and reduce cell proliferation in primary PBMC
Source: Sci Rep. 2017 Nov 30;7:16631. doi: 10.1038/s41598-017-15978-2 (PMC5709422; doi:10.1038/s41598-017-15978-2)

Low-dose levels of bisphenol A inhibit telomerase via ER/GPR30-ERK signalling, impair DNA integrity and reduce cell proliferation in primary PBMC

Corinna Herz[1$], Hoai Thi Thu Tran[1, 2$], Nina Schlotz[1], Karin Michels[3] and Evelyn Lamy[1]*

[1]Molecular Preventive Medicine, Institute for Prevention and Cancer Epidemiology,

Medical Center – University of Freiburg, Faculty of Medicine, University of

Freiburg, Germany, Germany, Elsässerstraße 2, 79110 Freiburg, Germany

[2]Pharmaceutical Bioinformatics, Institute of Pharmaceutical Sciences, Albert-Ludwigs-University, Hermann-Herder-Str.9, 79104 Freiburg, Germany

[3]Institute for Prevention and Cancer Epidemiology, Medical Center – University of Freiburg, Faculty of Medicine, University of Freiburg, Germany, Elsässerstraße 2, 79110 Freiburg, Germany

[$] These authors contributed equally to this work.

*corresponding author: Dr. Evelyn Lamy

Elsässerstrasse 2, 79110 Freiburg, Germany

email: evelyn.lamy@uniklinik-freiburg.de

phone: + 49 (0)761/270-77320

fax: + 49 (0)761/270-77340

Supplementary Materials and Methods

Antibodies

The following primary antibodies labelled with fluorophore were used for flow cytometry: CD3_APC, human (clone clone: BW264/56), CD3-FITC, human (cloneBW264/56), CD69-PE, human (clone: FN50), CD25-VioBright FITC, human (clone: 4E3), CD28-PE, human (clone 15E8), (Miltenyi Biotec, Bergisch Gladbach, Germany).

Analysis of surface markers using flow cytometry

PBMC (2×10^5) were stained for surface expression of cell markers using antibodies to phycoerythrin (PE)-labelled anti-human CD28 monoclonal antibody (mAb) and additionally stained with FITC-labelled anti-human CD3 mAb. PBMC were stained for surface expression of CD25 and CD69 using PE-labelled anti-human CD69 mAb together with FITC-labelled CD25 mAb and APC-labelled CD3 mAb. Subsequently the cells were analysed by flow cytometry using a FACSCalibur™ (BD Biosciences, Heidelberg, Germany).

Analysis of cell viability

Cell viability was determined using LIVE/DEAD® Fixable Far Red Dead Cell Stain Kit (Waltham, Massachusetts, USA) according to the manufacturer's instructions.

Supplementary Figure Legends

Supplement Figure S1: Representative scattergrams showing characteristics of PBMC in culture A) direct after isolation from blood B) 24h after 1st stimulation using CD2/CD3/CD28 beads C) after 21 days D) after 22 days (24h after 2nd restimulation).
An FSC/SSC-plot was made and all lymphocytes were gated (1). The lymphocyte population was copied to a SSC/CD3-scatterplot identifying T-cells (CD3+) (2). The T-cell gate was then copied to a CD25/CD69-scatterplot identifying activated T-cells (3).

Supplementary Figure S2: A) Representative scattergrams of CD3/CD28 surface expression of cultured cells direct after isolation from blood (day 0), 24h after 1st stimulation using CD2/CD3/CD28 beads (day 1), after 21 days in culture and after 22 days in culture (24h after 2nd restimulation).
An FSC/SSC-plot was made and all lymphocytes were gated (1). The lymphocyte population was copied to a CD3/CD28-scatterplot identifying (CD3+/CD28+).
B) Representative histogram of cell viability after 24h of stimulation with CD2/CD3/CD28 beads as determined using the LIVE/DEAD® Fixable Far Red Dead Cell Stain kit.

Supplementary Figure S3: The pictures depict original immunoblots of phosphorylated p38 and JNK (A) or phosphorylated ERK1/2 and whole protein ERK1/2 (B). β-actin was used as loading control. PBMC were treated to 1 nM BPA for 15 min and total lysate was subjected to immunoblotting.

Supplementary figures

Supplementary Figure S1

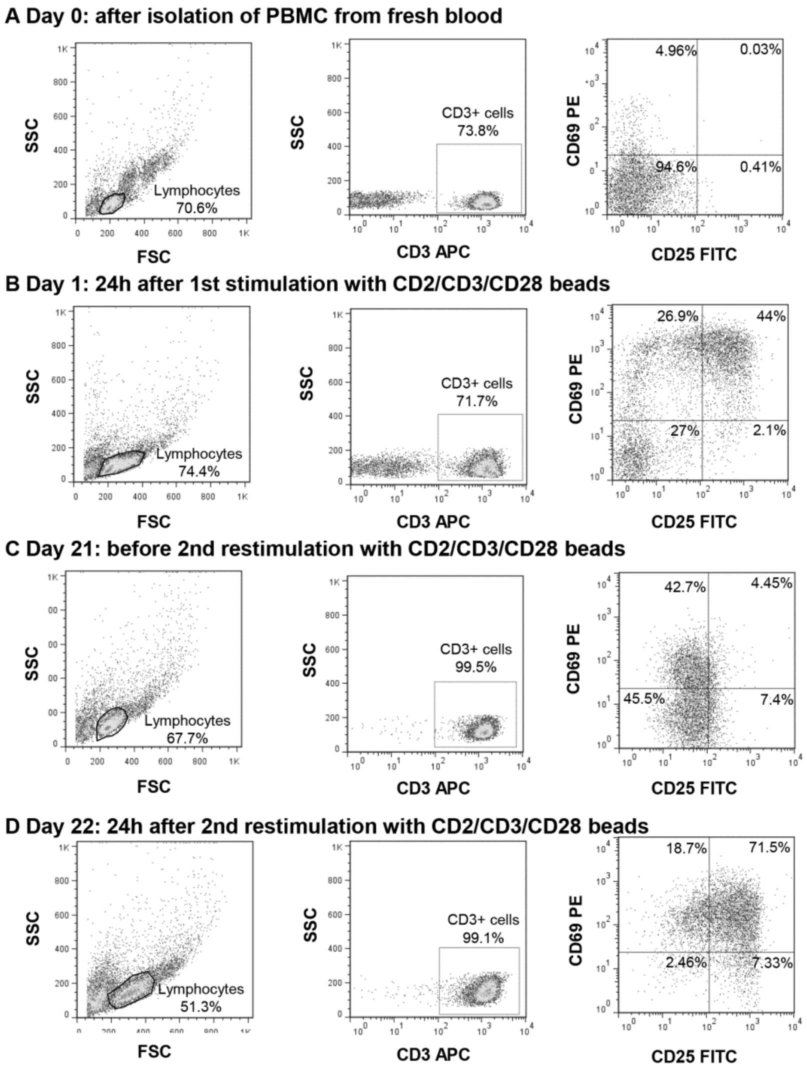

Supplementary Figure S2

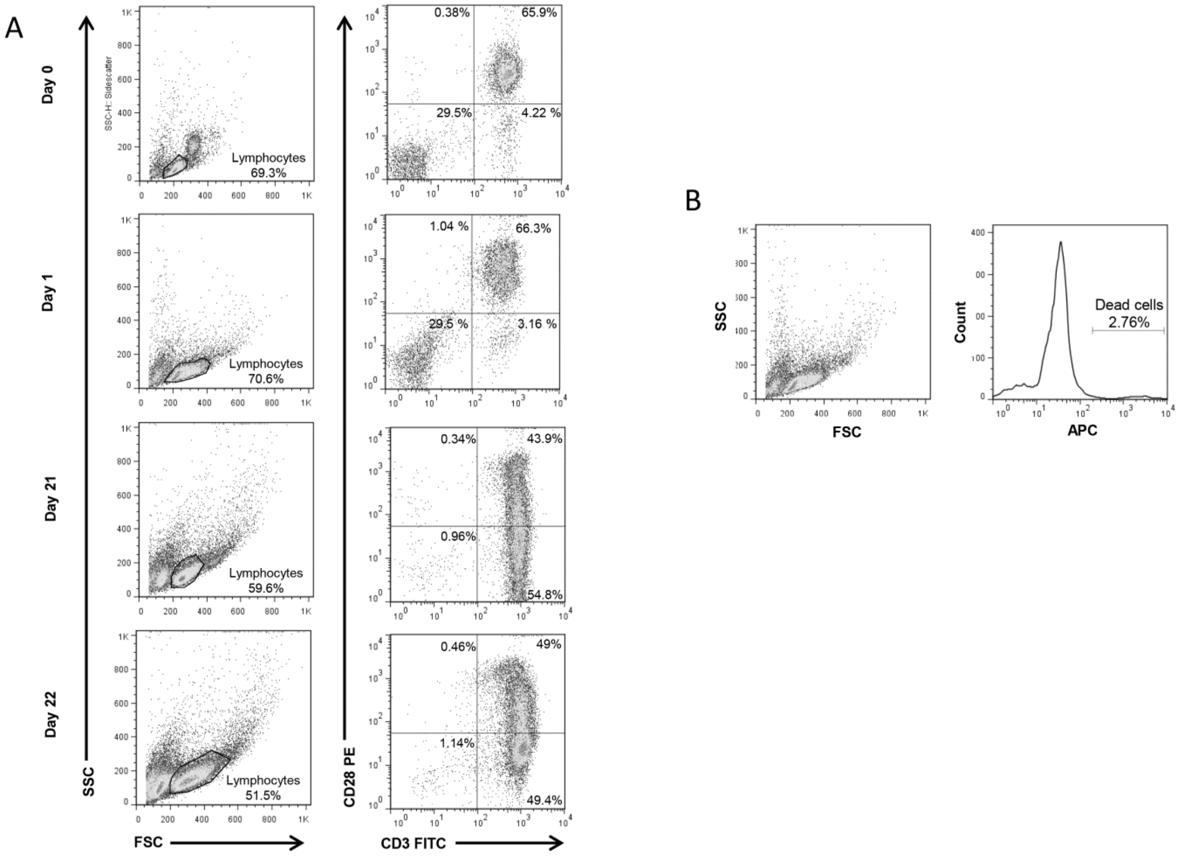

Supplementary Figure S3

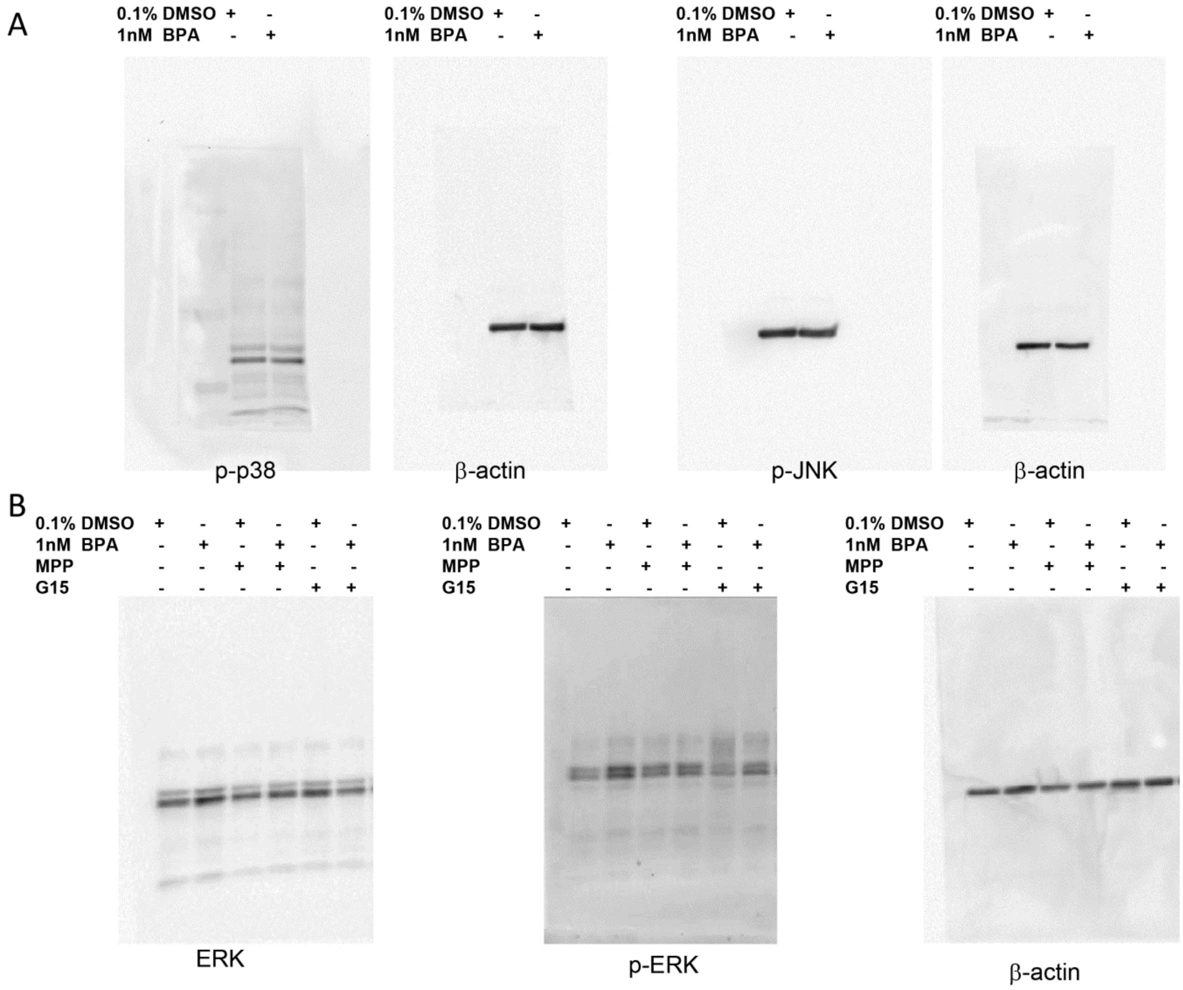

Supplement: Supplementary file 1 — Supplementary information [file 41598_2017_15978_MOESM1_ESM.pdf]
